# Human alteration of natural light cycles: causes and ecological consequences

**DOI:** 10.1007/s00442-014-3088-2

**Published:** 2014-09-20

**Authors:** Kevin J. Gaston, James P. Duffy, Sian Gaston, Jonathan Bennie, Thomas W. Davies

**Affiliations:** Environment and Sustainability Institute, University of Exeter, Penryn, Cornwall TR10 9FE UK

**Keywords:** Day, Diurnal, Night, Nocturnal, Skyglow

## Abstract

Artificial light at night is profoundly altering natural light cycles, particularly as perceived by many organisms, over extensive areas of the globe. This alteration comprises the introduction of light at night at places and times at which it has not previously occurred, and with different spectral signatures. Given the long geological periods for which light cycles have previously been consistent, this constitutes a novel environmental pressure, and one for which there is evidence for biological effects that span from molecular to community level. Here we provide a synthesis of understanding of the form and extent of this alteration, some of the key consequences for terrestrial and aquatic ecosystems, interactions and synergies with other anthropogenic pressures on the environment, major uncertainties, and future prospects and management options. This constitutes a compelling example of the need for a thoroughly interdisciplinary approach to understanding and managing the impact of one particular anthropogenic pressure. The former requires insights that span molecular biology to ecosystem ecology, and the latter contributions of biologists, policy makers and engineers.

## Introduction

Ecological systems are organized foremost by light, and particularly by daily and seasonal cycles of light and dark (Bradshaw and Holzapfel 2010; Kronfeld-Schor et al. 2013). Humans are profoundly altering these cycles as detected and/or perceived by many organisms. This is occurring by the introduction of artificial light at night (ALAN) in the environment, predominantly from electric lighting sources associated with human settlement, transport networks and industry, the impact of which extends across much of the globe (Cinzano et al. 2001). In turn this is influencing biological systems from the molecule to the ecosystem, including impacts on gene expression, physiology and behaviour of organisms, abundance and distribution of species, ecological interactions, and the composition of communities (e.g. recent examples include Bird et al. 2004; Davies et al. 2012; Dwyer et al. 2012; Dominoni et al. 2013a; Le Tallec et al. 2013; Mazor et al. 2013; Picchi et al. 2013). This then almost inevitably affects the function and process of ecosystems, and thus other fundamental ecological cycles. This paper reviews the form and extent of the human alteration of natural light cycles, key consequences for terrestrial and aquatic ecosystems, interactions and synergies with other anthropogenic environmental pressures, major uncertainties, and future prospects and management options. Several of these topics have not previously been well developed. As a synthesis, this is an illustrative rather than an exhaustive compilation of relevant studies, which are numerous but highly scattered within the literature (see Rich and Longcore 2006; Hölker et al. 2010; Perkin et al. 2011; Gaston et al. 2012, 2013; Bogard 2013; Gaston and Bennie 2014).

## Human alteration of natural light cycles

**Fig. 1:**
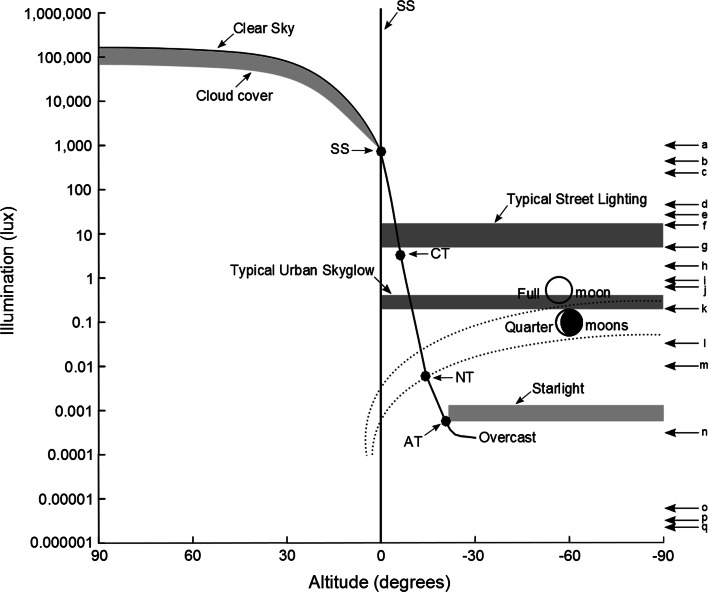
Change in illumination at the Earth’s surface with solar (*positive*) and lunar altitude (*negative*) above the horizon; typical illumination levels of artificial light at night (ALAN); and levels at which nighttime lighting has been observed to have biological effects [*arrows*; Sharma et al. (1997) (*a*), Zubidat et al. (2007) (*b*), Johnson (1979) (*c*), Stone et al. (2009) (*d*), Kuijper et al. (2008) (*e*), Riley et al. (2012) (*f*), Bedrosian et al. (2011) (*g*), Miller (2006) and Kramer and Birney (2001) (*h*), Falkenberg and Clarke (1998) and Clarke et al. (1996) (*h*), Santos et al. (2010) (*j*), Dauchy et al. (1997) and Cos et al. (2006) (*k*), Bachleitner et al. (2007) (*l*), Evans et al. (2007a) (*m*), Larsen and Pedersen (1982) and Dice (1945) (*n*), Dice (1945) (*o–q*); studies of levels at which nighttime lighting has biological effects are from Gaston et al. (2013, Table 3)]. Main figure modified from Beier (2006), with additional data from Kurtze (1974); Rich and Longcore (2006) and Gaston et al. (2013). *SS* Sunset, *CT* civil twilight, *NT* nautical twilight, *AT* astronomical twilight

**Fig. 2a–g:**
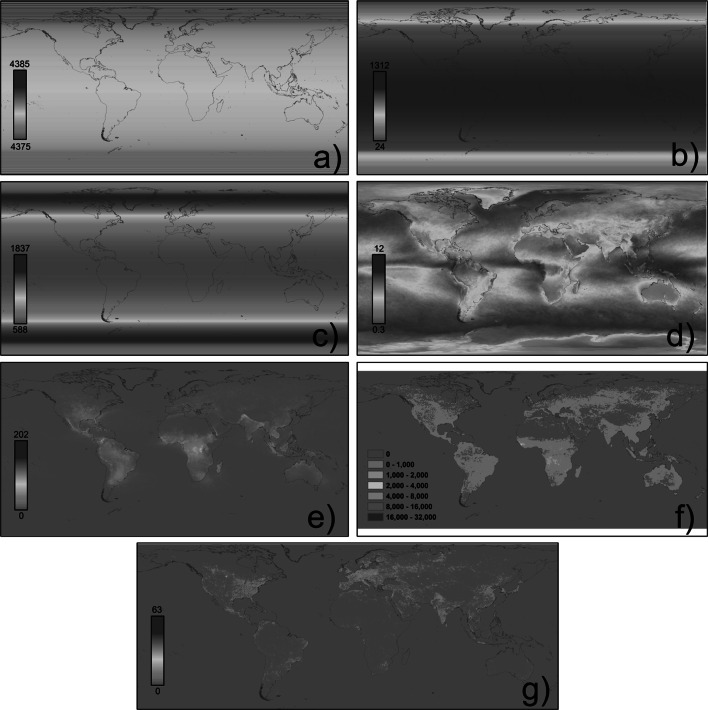
Global distributions of natural and artificial light. **a**–**c** Modelled yearly levels of daylight, moonlight and twilight in total hours, respectively, following equations in Meeus (2008), **d** cloud cover [composite of 12 monthly cloud fraction images for 2012 (Stockli 2013)], **e** mean annual lightning flash rate [flashes km^−2^ year^−1^ for 2012; Lightning Imaging Sensor/Optical Transient Detector gridded lightning climatology data set (NASA 2012)], **f** fire [sum of 12 cloud-corrected fire pixel images for 2012; NASA Land Processes Distributed Active Archive Center (2013)], and **g** artificial nighttime lighting {represented as digital number in 2010 [US Defense Meteorological Satellite Program (DMSP)/Operational Linescan System (OLS) 2012]}

Natural light cycles are, of course, driven entirely from an extraplanetary source, the sun. The primary cycles take three distinct forms (others with longer periods are not relevant here). First, rotation of the Earth partitions time into a regular cycle of day and night, such that the intensity of incident light at a site typically varies by ca. eight orders of magnitude (Fig. 1). Second, the Earth’s orbital motion and tilt of its axis cause marked seasonal variation in the distribution of the approximately 4,400 h of each year spent under conditions of relative darkness (including twilight, moonlight and starlight). Third, the nighttime light environment is subject to systematic variation as a consequence of the orbit of the moon around the Earth, and the level and pattern of reflected sunlight (moonlight) during the night. The effect of all three of these cycles on the light received at ground level (Fig. 2a–c) is modified to some degree by local topography, habitat and weather (especially cloud cover; Fig. 2d), and by other intermittent natural sources of light (e.g. lightning strikes and fires; Fig. 2e, f). However, the amplitude and frequency of these cycles prevail.

These daily, annual and lunar geophysical cycles have also remained rather invariant over long periods of time. For example, the Earth’s period of revolution around the Sun has been effectively constant. Its period of rotation around its axis, presently 24 h, has experienced deceleration, such that day length has increased through geological time, from ~21 h at the beginning of the Cambrian (Wells 1963). However, this amounts to a rate of increase only of ~0.002 s per century (Wahr 1988). This background means that ALAN is rather unusual amongst anthropogenic environmental pressures. Most others (e.g. changes in CO_2_, precipitation, temperature) have historical analogues, having previously altered naturally over geological or evolutionary time in similar ways to those presently experienced, albeit often at different rates. The most fundamental human-caused change to light cycles has two key characteristics, changes in the spatial and temporal occurrence of light and changes in its spectrum.

### Changing occurrence of light

**Fig. 3:**
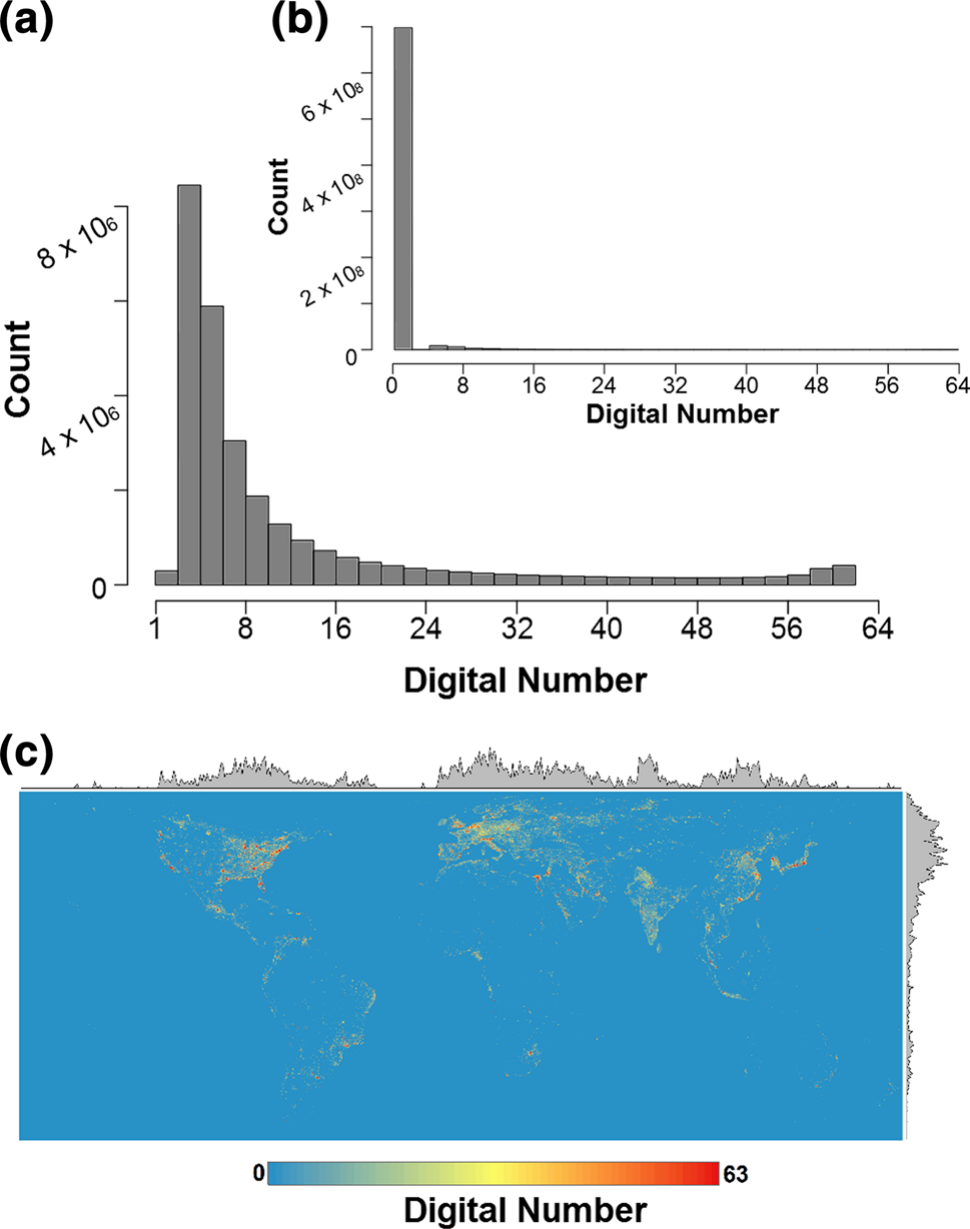
A raw nighttime stable lights image (2012) from the US DMSP/OLS (in Behrmann equal-area projection at a resolution of 810 × 810 m). Digital number indicates light intensity with 0 representing darkness and 63 indicating the brightest pixels. Histograms show data from this image **a** without and **b** with 0 digital number values. **c** *Grey graphs* show the mean digital number for particular columns/rows (latitudes/longitudes)

ALAN has introduced light in places, times and at intensities at which it does not naturally occur (Figs. 1, 2). The extent of these changes remains to be fully evaluated. Data principally arise from satellite imagery and aerial surveys of zenith-directed light emissions on cloud-free nights (e.g. Elvidge et al. 1999; Levin and Duke 2012; Miller et al. 2012; Mazor et al. 2013; Bennie et al. 2014a). Data from a recent satellite image, at a resolution of 810 × 810 m, indicate occurrence of ALAN in 3.4 % of cells globally (defined as a digital number >5), and 0.2 % of marine cells, but 11.4 % of terrestrial ones (Fig. 3). Where it does occur, this light varies markedly in intensity, even given saturation in the sensors at higher levels of emissions (Fig. 3). Spatial variation in the level of nighttime lighting tends to be well correlated with the level of development, built density, population density and economic activity of an area (Sutton 2003; Amaral et al. 2006; Doll et al. 2006; Chen and Nordhaus 2011; Pun and So 2011; Li et al. 2012; Hale et al. 2013). Recent decades have seen widespread increases in the number of spatially distinct lights and in the lit area, with changes particularly marked in Asia (Small and Elvidge 2011, 2013). These estimates focus on temporally persistent sources of light, and thus underestimate the overall scale of change.

Light emissions detected from satellite imagery and aerial surveys can bear complex relations to those experienced at ground level. Key features of ALAN on the ground include a marked degree of spatial heterogeneity, with maximum values of intensity in areas of direct illumination, complex patterns of shading on the ground surface due to the number and location of light sources, and large areas affected by lower intensity illumination from reflected and scattered light in the atmosphere. It varies markedly in intensity, with areas such as sports fields and parking lots often lit to illuminance values of several hundred lux or above, ground-level illuminance in the vicinity of street lights around 10–40 lux and reduced to <1 lux several metres away.

ALAN that is emitted or reflected upwards can be scattered by water, dust and gas molecules in the atmosphere, resulting in skyglow. Studies of sky irradiance have been made for individual sites, sets of sites, and cities (Kyba et al. 2011a, b; Biggs et al. 2012; Davies et al. 2013). Skyglow can be detected over a much wider area than direct artificial lighting—extending tens and perhaps hundreds of kilometres from the source—particularly because of the contribution of light that is emitted or reflected upward at relatively shallow angles to the horizontal (Crawford 2000). Local levels tend to be closely associated with prevailing land use, being greater in more highly developed areas and declining away from these (Garstang 1986; Crawford 2000; Biggs et al. 2012). It can attain levels of up to 0.2–0.5 lux (Kurtze 1974; Eisenbeis 2006), and under cloudy conditions in urban areas skyglow has been shown to be of an equivalent or greater magnitude than high-elevation summer moonlight (Kyba et al. 2011b). Indeed, cloud cover (which varies markedly; Fig. 2d) increases ALAN, the reverse of what happens during daytime (Kyba et al. 2011b). On clear nights skyglow reduces the visibility of stars and other celestial objects (Kyba et al. 2013).

Modelling techniques enable global sky brightness estimates to be obtained using satellite imagery of nighttime lights (e.g. Cinzano et al. 2001; Cinzano and Elvidge 2004). One such exercise estimated that at the turn of the twenty-first century about two-thirds of the global human population already lived in areas where sky brightness is above the threshold set for polluted status, about one fifth had lost naked-eye visibility of the Milky Way, and for about a tenth sky brightness was such they no longer viewed nighttime skies with the eye adapted to night vision (Cinzano et al. 2001).

### Changing spectra of light

**Fig. 4:**
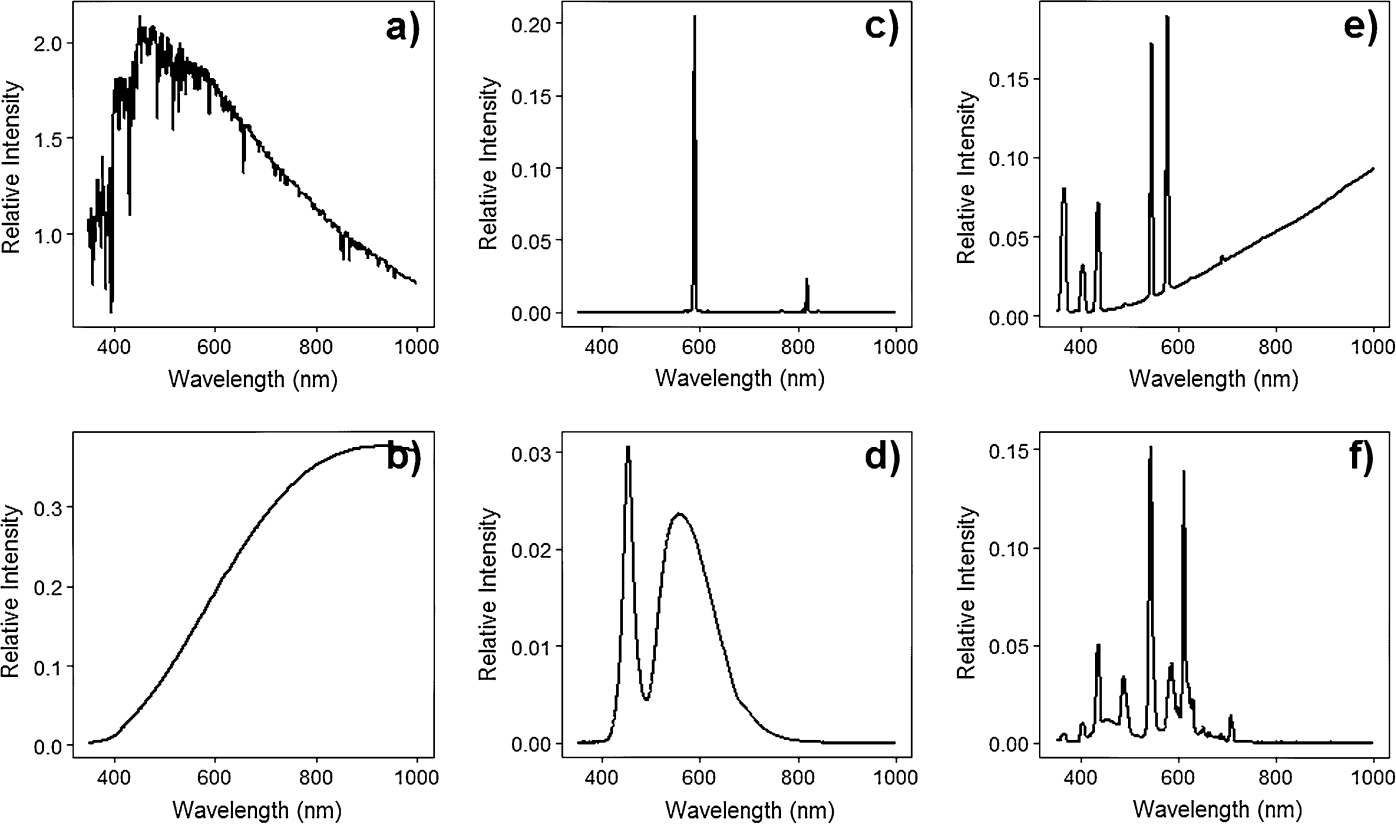
The spectral radiance of **a** daylight, **b** incandescent, **c** low-pressure sodium, **d** light-emitting diode, **e** mercury vapour, and **f** fluorescent lighting. Daylight spectra from pveducation.org and artificial light spectra from http://ngdc.noaa.gov/eog/night_sat/spectra.html

Not only does ALAN change the spatial and temporal structure and intensity of natural light cycles, it also occurs with spectra different from those of sunlight, moonlight or starlight (Fig. 4). Some types of artificial lighting are restricted to narrow bandwidths (e.g. low-pressure sodium lighting emits a single narrow peak in the visible spectrum at 589.3 nm). Others emit over a wide range of wavelengths [high-pressure sodium lighting emits a yellow light allowing some colour discrimination in humans; high-intensity discharge lamps emit a whiter light, with significant peaks in blue and ultra-violet wavelengths, and light-emitting diode (LED)-based white street lighting typically emits at all wavelengths between around 400 and 700 nm, with peaks in the blue and green (Elvidge et al. 2010)]. The prevailing sources tend to vary from one region to another, and hence the nature of the resultant ALAN. There is, however, a trend towards the adoption of lighting technologies with a broader spectrum of ‘white’ light. This increases the amount of skyglow visible to people and many other organisms (Van Tichelen et al. 2007, p. 91).

## Sources of change

There is a diversity of principal public and private sources of ALAN.

### Street lights

Street lighting appears from satellite and aerial imagery to be the dominant terrestrial source of ALAN, albeit not that with the most intense emissions (Kuechly et al. 2012). To some extent this is because street lights are more prone to upward unshielded or reflected light emissions, but it also results from the huge numbers of such lights and the lighting being unconstrained by other factors (e.g. lighting from within buildings is filtered through windows). Good estimates of the actual numbers of street lights appear to be lacking, although one recent figure suggests there are ca. 60 million in EU countries alone (Van Tichelen et al. 2007). However, the global paved road network, much of which is typically lit at night, is estimated at 18,015,713 km (CIA 2011), giving some indication of the potential extent of this source of ALAN.

### Buildings

The internal and, particularly, external lighting of buildings contributes substantially to nighttime views of major cities and conurbations, rendering some iconic in this regard (e.g. Paris, Las Vegas, Hong Kong, Shanghai). Urban areas are typically defined in terms of the level of coverage by buildings and associated infrastructure. Estimates of urban land cover are highly variable (Gaston 2010), but typical global figures are of the order of 2–3 % of land [excluding permanent ice cover (e.g. Millennium Ecosystem Assessment 2005)]. However, regional coverage may be substantially larger; figures for 165 countries vary from close to zero to 32 % (World Resources Institute 2007).

### Road vehicles

Terrestrially, the headlights of road vehicles produce substantial quantities of ALAN. On all but the busiest roads these emissions are temporally highly variable, occur predominantly in the horizontal plane, and are thus underestimated from satellite and aerial imagery. The orientation of these emissions means they may propagate over long distances. They have also progressively increased with major developments in headlight technology (Mainster and Timberlake 2003). Globally, in 2012 there were an estimated 833,342,000 passenger cars and 309,888,000 commercial vehicles (Organisation Internationale des Constructeurs d'Automobiles 2014), although it is unclear what proportion of these are used at night and with what frequency. The ecological impacts of ALAN from traffic has been little explored (Lyytimäki et al. 2012).

### Vessels

In the marine environment, significant ALAN is produced by shipping and offshore infrastructure such as oil and gas platforms. Particular attention has been paid to that generated by fishing fleets [especially those employing banks of lights to attract squid (e.g. Kiyofuji and Saitoh 2004; Elvidge et al. 2001)]. Although these lights are transient, much shipping is aggregated along common routes around coastlines and across oceans (Kareiva et al. 2007), and fishing fleets whilst operating over much larger extents tend disproportionately to focus activities on quite constrained areas (Jennings and Lee 2012).

## Effects on terrestrial ecosystems

**Fig. 5a–d:**
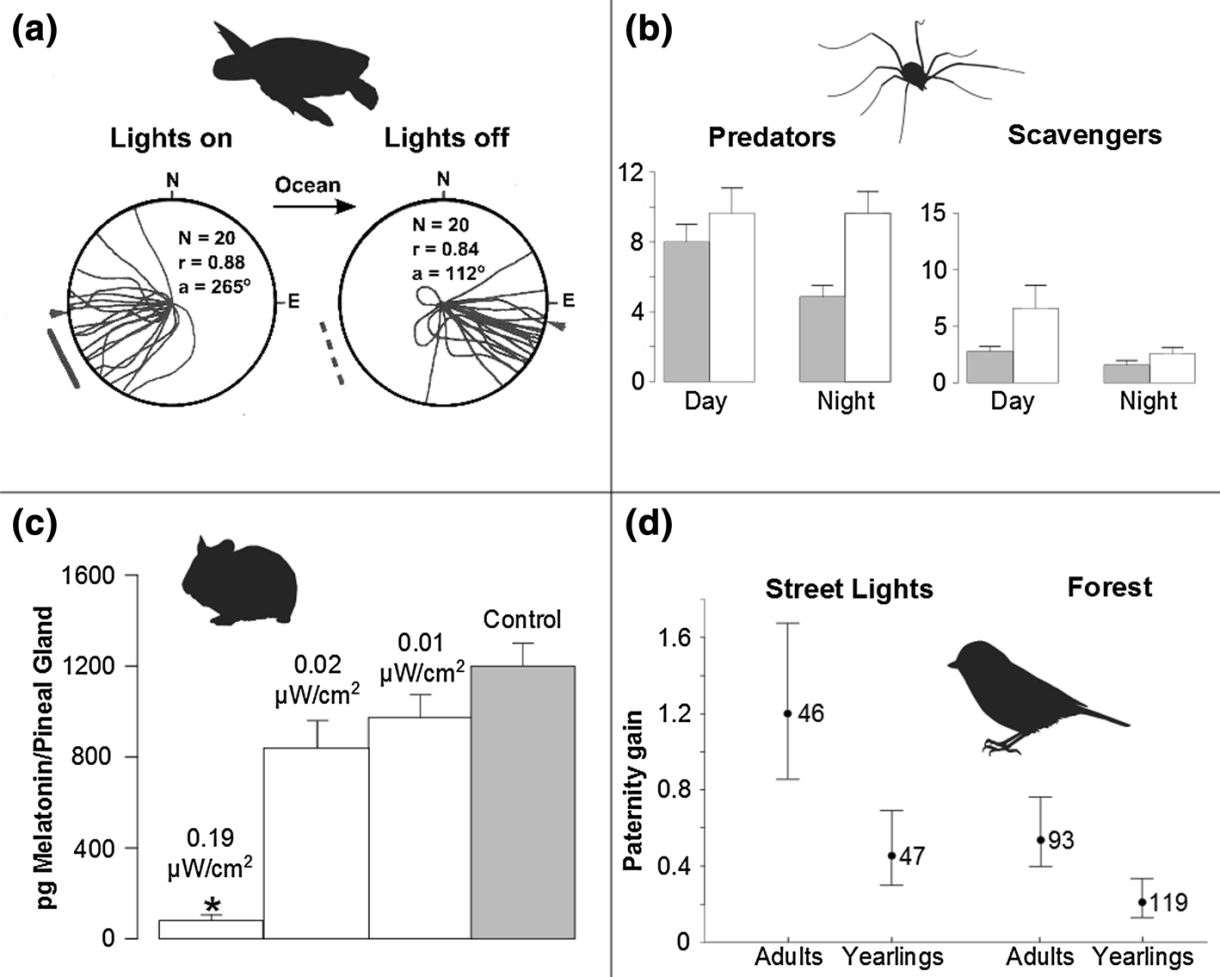
The effects of ALAN on animals. **a** Loggerhead turtle hatchlings crawl towards artificial light when it is turned on, and the ocean when it is turned off (Salmon et al. 1995). **b** The effect of high-pressure sodium street lighting on the abundance of invertebrates within trophic groups. *Bars* represent the total number of individuals in each group collected from pitfall traps under lights (*open bars*) and between lights (*grey bars*) (Davies et al. 2012). **c** The influence of light intensity on the suppression of pineal melatonin content after 30 min of exposure. *Bars* indicate mean pineal melatonin content (for each group *n* = 7). *p < 0.001 (Brainard et al. 1984). **d** The effect of artificial night light on paternity gain for adult and yearling blue tits *Cyanistes caeruleus* occupying edge territories; data are point estimates and 95 % confidence intervals from a generalised linear mixed model in which age and territory category are fixed factors and male identity and season are random intercepts;* numbers* show sample sizes (Kempenaers et al. 2010)

There have been a number of reviews of the ecological effects of ALAN, focusing principally on evidence of their breadth, in terms of different processes or levels of biological organization (Fig. 5; Longcore and Rich 2004, 2006; Gaston et al. 2013; Gaston and Bennie 2014), taxonomic levels (Rich and Longcore 2006) and research domain (Perkin et al. 2011). Here we highlight selected effects that in our opinion are emerging as likely to be of key significance in terrestrial and in aquatic ecosystems, starting with the former.

### Individual health

Natural light cycles influence the timings of numerous physiological processes. In many animals melatonin plays a key role in this (Vivien-Roels and Pévet 1993; Arendt 1998). Exposure either to even brief periods of high-intensity ALAN, or to prolonged periods of low intensity, has been shown in the laboratory to be capable of substantially altering patterns of circadian clock gene expression and melatonin production (e.g. Dauchy et al. 1997; Bedrosian et al. 2013; Schwimmer et al. 2014). In turn this can result in changes in expression of heat shock proteins, cortisol production and immune function, and increased risk of cancer (e.g. Dauchy et al. 1997; Bedrosian et al. 2011, 2013; Ashkenazi and Haim 2012; Schwimmer et al. 2014). This suggests that wild populations may also experience significant health impacts from ALAN. As yet, empirical evidence remains largely lacking, but so do studies whose goal is to obtain this evidence.

### Time partitioning

**Fig. 6a–d:**
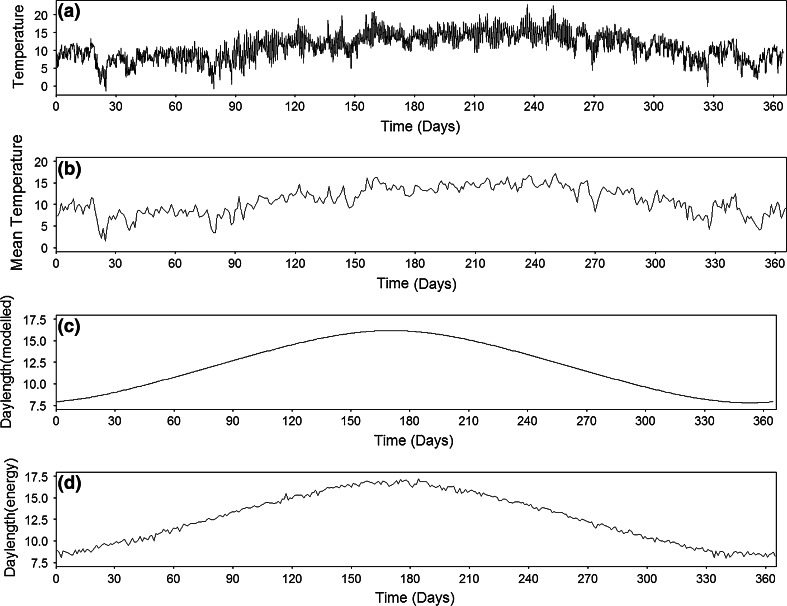
Actual temperature, and day length calculated in two different ways for 2007 at Penryn Campus, Cornwall. **a** Temperature (°C) recorded on a minute-by-minute basis, **b** mean daily temperature, **c** estimated day length based on latitude and the angle of the sun, and **d** estimated day length based on minute-by-minute energy measurements (kW m^−2^; beginning of day was identified as the time when values increased from 0 to >0 and end when they decreased from >0 to 0. Day length was then assumed to be the time between these two points). Data courtesy of K. Anderson

The timing of life history events is fundamental to fitness in perhaps most organisms (Bradshaw and Holzapfel 2010). Many use the timings of dawn and dusk, and/or day length as a cue for daily (e.g. foraging) and phenological events (e.g. growth, reproduction, migration), as in much of the world (foremost excepting environments with limited seasonal variation and where information is unreliable or inaccessible) these are more reliable than key alternatives (Fig. 6; such as temperature), although the latter may be used to modulate responses (e.g. Basler and Körner 2012; Saikkonen et al. 2012; Helm et al. 2013). ALAN has been found to cause changes in many such timings, including of singing (Nordt and Klenke 2013), activity (Boldogh et al. 2007; Dominoni et al. 2013b, 2014), foraging (Bakken and Bakken 1977; Bird et al. 2004; Lebbin et al. 2007), and births (Boldogh et al. 2007).

### Interspecific interactions

**Fig. 7:**
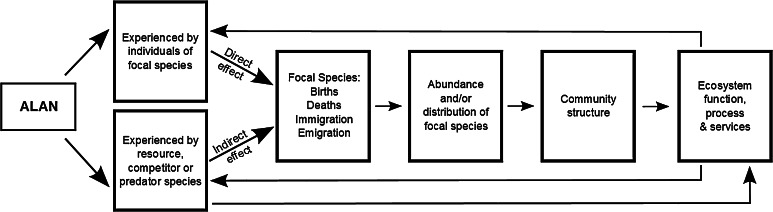
Routes by which influences of ALAN on interspecific interactions have consequences for community structure and ecosystem function, process and services

Diurnal and nocturnal species assemblages can differ markedly in their taxonomic and structural composition (e.g. Guevara and Avilés 2013; Bennie et al. 2014b). They typically comprise mixes of species that exhibit more obligate or more facultative time-partitioning behaviours (although most species probably have some flexibility), and there is some tendency for more detailed studies to reveal previously undocumented variation in time partitioning. The time-partitioning behaviour of animal species has been shown to be influenced by, and to influence, both competitive and predator–prey interspecific interactions (e.g. Fenn and Macdonald 1995; Schwartz et al. 2010; Pita et al. 2011). ALAN has thus been found to be able to change these interactions (e.g. Arlettaz et al. 2000). This can occur in two ways (Fig. 7). First, ALAN can directly influence the time partitioning of individuals of a focal species, with consequences for its interactions with others. Alternatively, ALAN can influence the time partitioning of competitors, prey or predators, causing in turn that of the focal species to change.

## Effects on aquatic ecosystems

The effects of ALAN on aquatic ecosystems, whether freshwater or marine, have been much less frequently studied than for terrestrial ecosystems (for reviews see Montevecchi 2006; Moore et al. 2006; Nightingale et al. 2006; Perkin et al. 2011; Davies et al. 2014). This is logical in as much as the majority of sources of ALAN are themselves terrestrial, and a greater proportion of the land mass is subject to ALAN than of the oceans. However, the high proportion of the global human population that is distributed close to major watercourses, lakes and along coasts (Small and Cohen 2004) suggests that some kinds of aquatic ecosystems may be disproportionately subject to ALAN (e.g. Aubrecht et al. 2008; Davies et al. 2014). Although many of the same influences are associated both with terrestrial and aquatic ecosystems, again, we highlight selected issues that are emerging, in our opinion, as likely to be of key significance for the latter.

### Reproduction

Natural light regimes, and notably lunar cycles, are used widely by marine organisms, either in isolation or in combination with other environmental cues, to time key reproductive activities. These organisms include polychaetes, cnidarians, echinoderms, and arthropods (e.g. Rudloe 1980; Lessios 1991; Tanner 1996; Bentley et al. 1999; Naylor 1999; Mercier et al. 2007; Harrison 2011). ALAN has significant potential to provide misleading information about when to time these reproductive activities, particularly for species reproducing in coastal waters. In turn, this could reduce synchrony of these activities amongst individuals (with consequences for fertilization success, predator satiation, etc.), and interactions with other important environmental phenomena, such as oceanographic processes and resource availability.

### Movements

Much attention has been paid to the influence of ALAN on the movements of organisms in terrestrial ecosystems (e.g. Frank 1988; Beier 1995; Gauthreaux and Belser 2006; Rydell 2006; Stone et al. 2009; Polak et al. 2011). However, patterns of light are arguably more important cues for movement in aquatic systems, where alternatives (e.g. use of landmarks) may often be severely lacking (Davies et al. 2014). Indeed, ALAN has already been shown to influence the movements (local, dispersive, migratory) of aquatic groups as diverse as zooplankton (Moore et al. 2000), fish (Ryer et al. 2009; Riley et al. 2012, 2013), turtles (Philibosian 1976; Lorne and Salmon 2007; Bourgeois et al. 2009) and birds (Telfer et al. 1987; Rodríguez and Rodríguez 2009; Rodrigues et al. 2012; Rodríguez et al. 2012a, b). Of particular concern is the extent to which ALAN impacts on the vertical diel movements of zooplankton, which are argued to constitute the largest synchronized movement of biomass globally, with huge impacts on carbon cycling and ecosystem functioning. These diel movements have been found to occur even during the polar night, regulated by variation in light intensity at levels below the threshold of human perception (Berge et al. 2009). This suggests that such movements may be highly susceptible to ALAN.

### Community structure

Effects of ALAN on births and deaths of species and/or their movements will result in shifts in community structure. Because the influences on demographic rates are likely to be site, time and species specific (Gaston and Bennie 2014), and to lead to shifts in competitive and predator–prey interactions, it is virtually impossible to predict a priori the form that these changes in community structure will take, and they are likely to appear quite idiosyncratic. Nonetheless, these changes have indeed been documented. For example, Meyer and Sullivan (2013) detail changes in the taxonomic and functional composition of aquatic and terrestrial invertebrate communities when natural streams were experimentally subjected to ALAN, reflecting changes in the fluxes between the two faunas. Likewise, Becker et al. (2013) document changes in the trophic and size structure of estuarine fish assemblages when artificial lighting conditions were manipulated. Given the links between community structure and composition and ecosystem functions and processes, ALAN will inevitably impact the latter, although to our knowledge these effects remain to be documented.

## Interactions and synergies

ALAN is, of course, only one of many anthropogenic pressures to which natural environments are subject, including habitat loss and fragmentation, climate change, excessive nutrient load and other forms of pollution, overexploitation and unsustainable use, and invasive alien species. One could potentially ask how ALAN compares in terms of the relative impact that it has. However, given that the different pressures seldom act in isolation it seems more pertinent to consider their interactions and synergies with ALAN. Here we highlight several such possibilities.

### Habitat loss and fragmentation

Most consideration of levels of habitat loss and fragmentation and their effects on ecosystems and biodiversity pertains to structural changes, such as in different kinds of land cover, in the physical sizes of patches, and in their degree of connectivity or isolation (Hanski 2005). ALAN can exacerbate these effects in ways that are not apparent from the daylight images (from aerial photographs and satellite sensors) that are typically employed to make such assessments. It renders areas of structurally unaltered habitat unusable by some organisms, available to others, and creates barriers to or corridors for movement that fragment and connect landscapes in different ways (Beier 1995, 2006; Eisenbeis 2006; Frank 2006; Stone et al. 2012; Threlfall et al. 2013). Indeed, full understanding of habitat loss and fragmentation needs to account both for diurnal and nocturnal effects, which may be rather different. Given the high proportion of species that are nocturnal (in addition to those that are crepuscular and cathemeral) in some groups of major conservation concern [e.g. 69 % of mammals (Bennie et al. 2014b)], it seems likely that the full impact of habitat loss and fragmentation has often been markedly underestimated.

### Climate change

It has previously been observed that biotic responses to anthropogenic climate change are critically dependent on the fact that whilst temperatures are changing, geographic and annual patterns in natural light cycles are not (Bradshaw and Holzapfel 2010). Given that organisms use day length as a cue for anticipating seasonal changes, this creates strong selection pressures for altering the timing of seasonal events, some of which they are able to respond to and some of which they are not (Bradshaw and Holzapfel 2010). ALAN serves to complicate this picture. Typically it serves locally to extend apparent day lengths, and to obscure their seasonal patterns. In combination, higher temperatures and increased light levels at night may allow species that are able to utilize the night light niche to extend their hours of activity (Garber 1978; Heiling 1999) and may alter predation patterns and/or competitive interactions.

### Other forms of pollution

ALAN can be seen as a stressor on the physiologies of many organisms, particularly as mediated through melatonin production. It seems likely that this will be more challenging to deal with in the presence of other forms of pollution, which are imposing other demands. ALAN can also exacerbate other forms of pollution in a more direct fashion. Stark et al. (2011) showed that artificial lights can change nighttime atmospheric nitrogen chemistry. Dim nocturnal light has also been found to inhibit recovery from leaf damage caused by atmospheric ozone in some species of clover *Trifolium* (Futsaether et al. 2009; Vollsnes et al. 2009).

### Overexploitation and unsustainable use

The harvesting of many marine species (e.g. shrimp, squid, fish) employs the use of artificial nighttime lights as attractants, sometimes on an industrial scale (Kiyofuji and Saitoh 2004). The effects of this source of ALAN on unexploited organisms is largely unknown; however, it seems likely to be potentially marked, particularly given the responsiveness of most marine organisms to light. Some forms of terrestrial harvesting, such as spotlighting, also employ ALAN, but this is on a more localized and transient scale.

### Invasive alien species

Several examples exist of invasive alien species that have rapidly adapted photoperiodic responses to their new environment [over a few decades (Gomi and Takeda 1991; Urbanski et al. 2012)]. The ability to adapt phenology to changing light regimes may be a key determinant of success in colonizing latitudes outside of a species’ historical range (Bradshaw and Holzapfel 2010), which is critical when species are introduced to new regions or spread due to climate change. Such phenotypic flexibility in photoperiodism may also be important in species response to extended hours of light due to ALAN.

## Major uncertainties

Although the potential for ALAN to have significant biological impacts has long been recognized and a large body of studies has ensued (Rich and Longcore 2006; Gaston et al. 2013), substantial uncertainties remain. We would highlight the following as being, in our view, particularly significant gaps in knowledge:

1. What are the biological effects of skyglow resulting from ALAN? Whilst the effects of more direct lighting are increasingly well understood, those of skyglow remain poorly explored. Studies to do so are challenging, although suggestions as to how these might be constructed have been made (Kyba and Hölker 2013).
2. What are the effects of ALAN on photosynthesis? The widespread use of artificial lighting in growing plants under controlled conditions suggests the potential for ALAN to influence photosynthesis. However, studies of these impacts, and of those on the photophysiology of plants and phytoplankton more broadly, remain scarce and it is difficult to extract any broad conclusions (Gaston et al. 2013; Poulin et al. 2013).
3. Do the influences of ALAN on stress and disease demonstrated for animals in the laboratory extend to the wild? Particularly because of concerns about effects of ALAN on human health, more laboratory studies of potential ecological relevance have been conducted than for most ecological issues. However, there are undoubtedly large differences between the ALAN treatments used in laboratory settings and what the majority of organisms experience in the field, especially when those organisms are mobile.
4. What shape are dose–response curves for ALAN? The literature on the ecological effects of ALAN is dominated by studies in which comparison is made, observationally or experimentally, between the state of a given ecological variable with and without ALAN, or perhaps with two different forms of ALAN (usually differing in intensity, but sometimes light spectrum). Almost nothing is known about the form of dose–response curves for ALAN, and thus critically how responses are likely to change when ALAN attains different levels.
5. What are the impacts of ALAN on ecosystem functions and processes? Broadly speaking, most is known about the impacts of ALAN on the physiology and behaviour of organisms, less about those on population dynamics, little about those on communities, and almost nothing about the impacts on ecosystem functions and processes. Given that ALAN can influence the abundances of species and trophic interactions there seems little doubt that such effects on ecosystem functions and processes do occur.

## Future prospects and management options

ALAN will undoubtedly continue to spread globally, particularly given the rapid rate of population growth and industrialization in many countries (Small and Elvidge 2013), although the pattern may be more complex in industrialised regions (Bennie et al. 2014a). However, it will also alter in form, as the predominant technologies employed change. From an ecological perspective key changes include increasing use of:

1. Central management systems in developed countries by which the timing and intensity of grid-based lighting can be controlled, already resulting in some broad-scale decreases in lighting during periods when it is not needed (Bennie et al. 2014a);
2. White light technologies, especially LEDs. LEDs can be modified to control the spectral composition of lighting, can require lower wattage for a given level of illumination than more traditional light sources, provide high light output for low radiant heat, can distribute light more uniformly and thus allow lower levels of lighting to be employed, are dimmable and more tolerant of switching on and off, and have long life times before failure (US Department of Energy 2012). Typical white LEDs emit considerably more light in the blue portion of the spectrum than conventional ‘white’ lighting (Fig. 4); while LED technology may allow more control over the spectra emitted, a movement towards white LED-based lighting systems is likely to lead to greater emissions within the blue portion of the spectra. LEDs also raise concerns around hazardous waste and resource depletion (Lim et al. 2011).
3. Off-grid lighting in developing countries, likely principally using combinations of LEDs and solar power (Mills 2005).

Various management options have been highlighted by which the ecological impacts of ALAN can be limited and/or reduced (Falchi et al. 2011; Gaston et al. 2012). In brief, these are:

1. Maintaining and creating dark areas. Faced with progressive loss of dark areas, particularly in more heavily urbanized regions it is important to protect those that remain and where possible recover others. There are a number of initiatives to identify presently dark areas, to highlight this status, and to encourage steps by which it is maintained [UNESCO 2009; International Dark Sky Association (IDSA) 2013; IUCN 2013]. There are also initiatives to encourage communities to reduce their overall levels of ALAN (IDSA 2001). Even quite localized changes (e.g. switching off a few key lights) can serve to reduce particular impacts (e.g. Yurk and Trites 2000).
2. Reducing light trespass. Lighting devices generally remain quite poorly designed and/or managed for the purposes of only directing light where it is actually required. Resolving this problem provides a ready means of dramatically decreasing the impacts of ALAN at a local scale. Indeed, reduction of light trespass has been shown to reduce the impacts of ALAN on organisms (e.g. Reed et al. 1985).
3. Dimming. Many areas are overlit compared with what is practically required. This provides opportunities for dimming of lighting without major negative consequences for human populations. Indeed, substantial progressive dimming may be possible without these populations being able to perceive that this is the case. The introduction of LED lighting provides further opportunities, given that colour rendering may be improved at lower intensities of lighting.
4. Part-night lighting. Many areas are presently lit at times of day when this carries limited or negligible human benefit. Particularly following the global financial crisis, and pressure on public expenditure, numerous towns and cities have sought to reduce energy costs (and CO_2_ emissions) by switching off street lights in low-risk areas from late at night until the early hours of the morning (Gaston et al. 2012). The ecological benefits of such part-night lighting remain poorly understood, and may only influence a relative minority of species that use the heart of the night rather than hours around dusk and dawn.
5. Targeting spectra. There are doubtless substantial opportunities to reduce the ecological impacts of ALAN by employing spatially more nuanced approaches to the use of lighting with different spectral properties. Developing alternatives to presently installed systems, which have often evolved as different technologies have become available and affordable, will require balancing of multiple pressures. These include cost, practicality, human perceived and actual need, and environmental concerns. In general, there would seem to be a number of advantages to the use of reddened spectra in environmentally more sensitive areas because, relative to white or blue sources, these reduce skyglow (Kyba et al. 2012), penetrate the water column to a lesser extent, have less influence on melatonin levels and circadian rhythms of species (Bayarri et al. 2002; Lockley et al. 2003), are less attractive to some organisms (e.g. Evans et al. 2007b; Cowan and Gries 2009; Somers-Yeates et al. 2013) and less repellent to others (e.g. Downs et al. 2003, Widder et al. 2005). However, this is not always the case—reddened light may disrupt the magnetic orientation of migratory birds (Wiltschko et al. 1993) and light of lower wavelengths may be less disruptive to these species (Poot et al. 2008). Furthermore, reddened light sources have a stronger influence on plant development through the impact on phytochromes, which respond to the ratio of red to far red light (Stutte 2009).

Arguably, there is a trade-off between the economic costs and the perceived social costs associated with implementing these different strategies to managing ALAN. This constitutes the major challenge to limiting its ecological impacts.
